# Dispersing and Sonoporating Biofilm-Associated Bacteria with Sonobactericide

**DOI:** 10.3390/pharmaceutics14061164

**Published:** 2022-05-30

**Authors:** Kirby R. Lattwein, Inés Beekers, Joop J. P. Kouijzer, Mariël Leon-Grooters, Simone A. G. Langeveld, Tom van Rooij, Antonius F. W. van der Steen, Nico de Jong, Willem J. B. van Wamel, Klazina Kooiman

**Affiliations:** 1 Department of Biomedical Engineering, Thoraxcenter, Erasmus MC University Medical Center Rotterdam, Office Ee2302, P.O. Box 2040, 3000 CA Rotterdam, The Netherlands; inesbeekers@gmail.com (I.B.); j.kouijzer@erasmusmc.nl (J.J.P.K.); h.leonmorales-grooters@erasmusmc.nl (M.L.-G.); s.a.g.langeveld@erasmusmc.nl (S.A.G.L.); tvrooij@gmail.com (T.v.R.); a.vandersteen@erasmusmc.nl (A.F.W.v.d.S.); n.dejong@erasmusmc.nl (N.d.J.); k.kooiman@erasmusmc.nl (K.K.); 2 Laboratory of Acoustical Wavefield Imaging, Faculty of Applied Sciences, Delft University of Technology, Building 22, Room D218, Lorentzweg 1, 2628 CJ Delft, The Netherlands; 3 Department of Medical Microbiology and Infectious Diseases, Erasmus MC University Medical Center Rotterdam, Office Na9182, P.O. Box 2040, 3000 CA Rotterdam, The Netherlands; w.vanwamel@erasmusmc.nl

**Keywords:** antibiotic, bacteria, biofilm, dispersion, microbubble, sonobactericide, sonoporation, *Staphylococcus aureus*, ultrasound

## Abstract

Bacteria encased in a biofilm poses significant challenges to successful treatment, since both the immune system and antibiotics are ineffective. Sonobactericide, which uses ultrasound and microbubbles, is a potential new strategy for increasing antimicrobial effectiveness or directly killing bacteria. Several studies suggest that sonobactericide can lead to bacterial dispersion or sonoporation (i.e., cell membrane permeabilization); however, real-time observations distinguishing individual bacteria during and directly after insonification are missing. Therefore, in this study, we investigated, in real-time and at high-resolution, the effects of ultrasound-induced microbubble oscillation on *Staphylococcus aureus* biofilms, without or with an antibiotic (oxacillin, 1 μg/mL). Biofilms were exposed to ultrasound (2 MHz, 100–400 kPa, 100–1000 cycles, every second for 30 s) during time-lapse confocal microscopy recordings of 10 min. Bacterial responses were quantified using post hoc image analysis with particle counting. Bacterial dispersion was observed as the dominant effect over sonoporation, resulting from oscillating microbubbles. Increasing pressure and cycles both led to significantly more dispersion, with the highest pressure leading to the most biofilm removal (up to 83.7%). Antibiotic presence led to more variable treatment responses, yet did not significantly impact the therapeutic efficacy of sonobactericide, suggesting synergism is not an immediate effect. These findings elucidate the direct effects induced by sonobactericide to best utilize its potential as a biofilm treatment strategy.

## 1. Introduction

Life-threatening biofilm infections pose a major challenge to healthcare systems, leading to substantial increases in mortality and expenses [1,2]. In addition to being community-acquired, 5–10% of all acute-care hospital patients admitted will develop a bacterial infection [1]; an estimated 65% of all, as well as 80% of recurrent and chronic infections, are associated with biofilms [3,4]. Biofilm infections occur when bacteria adhere to tissue and/or devices and subsequently encase themselves in a protective matrix as they grow. In addition to the increasing rates of bacteria with antibiotic resistance, biofilms hinder antibiotic effectiveness up to 1000-fold, compared to bacteria in a planktonic (free-floating) state [5]. Therefore, these patients often require long-duration, high-dose combination antibiotic therapy. When antibiotic failure occurs, local and systemic complications can quickly develop, often requiring surgery, without the guarantee of eradication [3,6]. A therapeutic strategy, either as an adjunct to enhance antibiotic efficacy or alternative to directly killing bacteria or breaking up biofilms, would be a major breakthrough for biofilm-associated infections.

One potential antibacterial strategy that is emerging in preclinical studies is sonobactericide [7]. It uses ultrasound and cavitation nuclei, such as phospholipid-coated microbubbles, combined with or without antibiotics and/or other therapeutics, for bacterial infection management. The ability of ultrasound and microbubbles to induce a bactericidal effect stems from microbubble oscillation, i.e., the expansion and contraction of the gas core induced by each ultrasonic wave. The oscillations, microbubble displacement, jetting, or microstreaming can directly exert a mechanical pressure on nearby cells and lead to other cell-affecting phenomena, such as reactive oxygen species production [8,9]. Microbubble-induced cellular effects have been extensively studied in mammalian cells and continue to be studied, as mechanistic pathways and microbubble behavior are not fully understood [9]. One such microbubble–cell effect is the enhancement of drug uptake pathways by stimulating endocytosis, opening cellular junctions, and increasing membrane permeability by membrane perforation, which is referred to as sonoporation [9,10,11]. Sonoporation causes cellular responses (ion in- and outflux, structural modifications, etc.) that alter cells and can result in cell death [12,13,14]. This and other mammalian cellular effects due to ultrasound-driven cavitation may also occur in bacterial cells, though most likely to different extents, given the many distinctive differences between eukaryotic and prokaryotic cells, such as high turgor pressures (internal osmotic pressure, e.g., ~5–25 atm [15,16]) and cell walls, which are present in bacteria and absent in mammalian cells. 

Sonobactericide has been demonstrated in several papers on *Staphylococcus aureus* (*S. aureus*) biofilms [17,18,19,20,21,22,23,24,25], which is the predominate infecting microbe for many types of infections, and it is associated with more severe disease, higher mortality, and longer hospital stays [26]. A range of effects, as a result of sonobactericide on *S. aureus* using non-targeted microbubbles, have been observed or suggested, including biofilm disruption/dispersal [19,21], sonoporation (not quantified) [23], and enhanced antimicrobial efficacy [19,21,23,24]. However, these studies did not provide real-time observations, both during and directly following insonification, and/or high-resolution imaging to distinguish individual bacteria; additionally, only one [21,23,24] or two [19] sets of acoustical parameters were utilized within an experimental set-up. Combining both real-time and high-resolution live-cell fluorescence imaging, while varying multiple acoustics parameters, would provide valuable information to better understand how sonobactericide works and could succeed as a therapeutic infection intervention. Therefore, in this in vitro study, the real-time responses of *S. aureus* in biofilms to ultrasound-induced microbubble oscillations were investigated at high-resolution, with a focus on sonoporation and cell dispersion. Ultrasound, at a frequency of 2 MHz, was used to insonify the biofilms with and without non-targeted microbubbles and/or the corresponding clinically relevant antibiotic oxacillin. Ultrasound pressures and cycles were varied, in order to determine whether the changes in either parameter affected the treatment outcome. Continuous confocal microscopy recordings of 10 min, combined with subsequent fluorescent image analysis, were used to assess the direct effects of sonobactericide on *S. aureus* biofilms.

## 2. Materials and Methods

### 2.1. Bacterial Isolate Handling and Characterization

The clinical *S. aureus* strain used in this study was isolated from a patient with confirmed infective endocarditis at the Erasmus University Medical Center Rotterdam, the Netherlands. The isolate was de-identified and anonymized according to institutional policy and stored at −80 °C. All overnight cultures were grown on blood agar plates (tryptic soy agar with 5% sheep blood; BD, Trypticase^TM^, Thermo Fisher Scientific, Waltham, MA, USA) at 37 °C. The antibiotic sensitivity profile of the isolate and bacterial species identification was determined using the VITEK 2 system (bioMérieux, Marcy-l’Étoile, France). 

The isolate was sent to the University Medical Center Groningen, Genome Analysis Facility (Groningen, the Netherlands) for whole-genome sequencing. The obtained genome data was analyzed to identify known *S. aureus* genes using the core-genome multi-locus sequence typing (MLST+) scheme in the BioNumerics 7.6.3 software (Applied Maths, Sint-Martens-Latem, Belgium) [27]. Additional genetic characterization with Staphylococcal protein A (*spa*) typing was performed as previously described by Harmsen et al. [28]. The raw sequencing data was analyzed with the BioNumerics software using the *spa*-typing plugin (Applied Maths). Both typing results were used in PubMed to search for disease association and other related information published in scientific literature.

### 2.2. Biofilm Formation

Biofilms were formed in vitro, as previously described [29], with minor modifications. Briefly, single bacterial colonies from overnight *S. aureus* cultures were suspended in 4 mL of saline solution (0.9% NaCl), in order to reach an optical density of 0.5 (±0.05) at 600 nm in a cell density meter (Ultraspec 10, Amersham Biosciences, Little Chalfont, UK). The suspension (0.5 mL) was then used to inoculate 9.5 mL of sterile Iscove’s modified Dulbecco’s medium (IMDM; Gibco, Bleiswijk, The Netherlands) in an acoustically compatible OptiCell^TM^ cell culture chamber (Nunc^TM^, Thermo Fisher Scientific, Wiesbaden, Germany). Inoculated OptiCells were placed in an incubator at 37 °C for 24 h, first statically for 3 h, in order to allow for bacterial adherence to the gas-permeable, polystyrene film bottom membrane, and then dynamically on a rotary platform shaker at 150 rpm (Rotamax 120, Heidolph Instruments, Schwabach, Germany). 

### 2.3. Biofilm Antibiotic Response Assay

To rule out that the results of the treatments with an antibiotic were, in fact, due to ultrasound and microbubble effects and not to the antibiotic alone, a time-lapse antibiotic killing curve experiment was performed. Biofilms were formed following the same protocol for the OptiCell, but volume scaled for black, 96-well, clear flat-bottom plates (Costar no. 3904; Corning Inc., Corning, NY, USA) by adding 10 μL of inoculate (0.5 optical density) to 190 μL IMDM in each well [29,30]. 

Following 24 h incubation, biofilms were gently washed thrice with 200 μL IMDM to remove planktonic cells. Propidium iodide (PI) was used as a fluorescence indicator of cell viability, since it is impermeable to living cells and can only bind to nucleic acids if the cell membrane is compromised, either from death or extrinsic factors [31]. Triton X-100 is a potent detergent that disrupts bacterial cell membranes, leading to cell lysis; thus, it was used for the positive control [32]. After washing, 200 μL containing IMDM and PI (25 μg/mL; P4864, Sigma-Aldrich, St. Louis, MO, USA) was added to the wells, either alone, with the antibiotic oxacillin (28221; Sigma-Aldrich) at concentrations ranging serially from 0.016 to 256 μg/mL, or with 0.1% Triton X-100 (X100, Sigma-Aldrich). The plates were then directly placed in a temperature-regulated microplate reader (FLUOstar Optima, BMG Labtech, Ortenberg, Germany), maintained at 37 °C, with shaking (150 rpm) between the automated plate fluorescence readings (excitation 541 nm, emission 612 nm) performed every 15 min for 12 h. 

### 2.4. Microbubble Preparation 

Non-targeted microbubbles, consisting of a phospholipid monolayer shell and C_4_F_10_ (F2 Chemicals, Preston, UK) gas core, were made using a 20-kHz ultrasonic probe (1 min sonication), as previously published [14,33]. The phospholipid shell was comprised of the following: 1,2-distearoyl-*sn*-glycero-3-phosphocholine (DSPC; 84.8 mol%; P6517; Sigma-Aldrich), polyoxyethylene-(40)-stearate (PEG-40 stearate; 8.2 mol%; P3440; Sigma-Aldrich), and 1,2-distearoyl-*sn*-glycero-3-phosphoethanolamine-N-carboxy (polyethylene glycol) (DSPE-PEG(2000); MW 2000; 7.0 mol%; PEG6175.0001; Iris Biotech GmbH, Marktredwitz, Germany). Prior to sonication, the coating components were dispersed in phosphate-buffered saline (PBS) saturated with C_4_F_10_ and 1,1′-diocta-decyl-3,3,3′,3′-tetramethylindodicarbocyanine perchlorate (DiD; D307, Thermo Fisher Scientific); a lipophilic, far-red fluorescent dye, was added to fluorescently label the microbubble shells. Microbubbles were washed three times by centrifugation (400× *g*) for 1 min in C_4_F_10_-saturated PBS, in order to remove excess dye and lipid debris before experiments. A Coulter Counter Multisizer 3 (Beckman Coulter, Mijdrecht, The Netherlands) with a 50 μm aperture tube was then used to determine the microbubble size distribution (range 1–30 μm) and concentration before experiments on the days using microbubbles (N = 10). The mean microbubble size was found to be 4.0 ± 0.3 μm (average ± SD).

### 2.5. Experimental Sonobactericide Set-Up 

**Figure 1:**
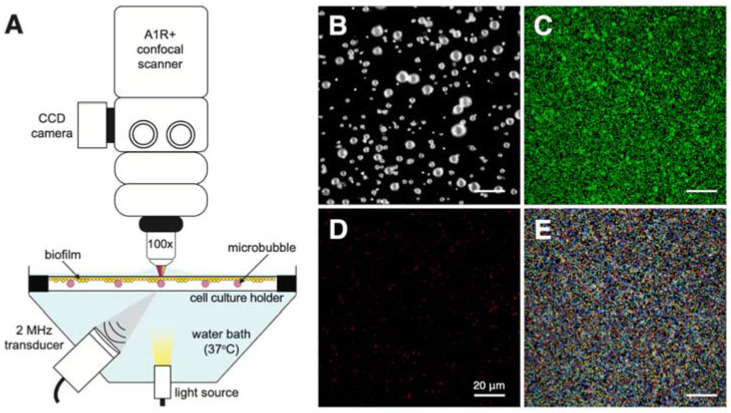
Experimental set-up and imaging example. (**A**) Schematic (not drawn to scale) of the experimental set-up, allowing for simultaneous optical imaging and ultrasound insonification, with the bacterial biofilm and microbubbles in the cell culture holder, i.e., OptiCell. Confocal image example of all three channels, where (**B**) microbubbles are fluorescently labelled with DiD lipid dye (pseudo-colored white), (**C**) living bacteria stained with SYTO 9 (pseudo-colored green), and (**D**) dead/membrane-compromised bacteria stained with propidium iodide (pseudo-colored red). (**E**) Image output of cells from (**C**), segmented in different colors and used for quantitative analysis. All scale bars represent 20 μm.

To allow for the high spatial resolution necessary to capture the micrometer cellular responses of *S. aureus*, a custom-built, upright Nikon A1R+ confocal microscope, with the same specifications as described previously [34], was used. An OptiCell was placed, with the biofilm on the upper membrane, into a temperature-controlled (37 °C) water bath, situated directly beneath the microscope, as depicted in Figure 1A. A single-element, focused ultrasound transducer (a 2.25 MHz center frequency used at 2 MHz, 76.2 mm focal distance, −6 dB beam width of 3 mm; V305 Panametrics-NDT, Olympus, Waltham, MA, USA), with an output that had been previously calibrated using a 1 mm needle hydrophone (PA2293; Precision Acoustics, Dorchester, UK), was situated below the OptiCell at a 45° angle, in order to minimize standing wave formation and buildup. The acoustical focus was aligned with the optical focus for simultaneous ultrasound insonification and visualization. The insonification protocol consisted of a 2 MHz acoustic pulse, with either a 100 or 1000 cycle burst, at a pulse repetition frequency (PRF) of 1 Hz for 30 s, generated by an arbitrary waveform generator (DG1032Z; RIGOL Technologies Inc., Beijing, China). A 2 MHz frequency is within the frequency range used by diagnostic scanners and transthoracic echocardiography [35,36]. The signal was amplified (ENI A-500 broadband amplifier; Electronics & Innovation, Rochester, NY, USA) to obtain peak-negative pressures of 100, 200, and 400 kPa at the focus, for an overall MI < 0.3. The confocal microscope was equipped with a 100× water dipping objective (CFI Plan 100XC W, 2.5 mm working distance, Nikon Instruments, Amsterdam, the Netherlands) for all optical imaging.

### 2.6. Experimental Sonobactericide Protocol

Five different experimental ultrasound insonifications were used: no ultrasound; 100 kPa, 100 cycles; 100 kPa, 1000 cycles; 200 kPa, 100 cycles; and 400 kPa, 100 cycles. This was performed with and without microbubbles, as well as with and without oxacillin, on a total of 26 biofilms, over 13 experimental days (total of 176 experimental exposures). If the experimental arm included oxacillin, a concentration of 1 μg/mL was used, because it is above the minimal inhibitory concentration of ≤0.25 μg/mL and below the peak serum concentration of 43 μg/mL reported for 500 mg slow IV administration, taking into consideration the 94% protein binding [37]. Oxacillin was added to biofilms 3.5 h before experiments, based on the antibiotic response assay, indicating that the PI uptake due to oxacillin alone was minimal and stable after this time point.

Before each OptiCell was placed into the water bath, the *S. aureus* within the biofilm were fluorescently stained to visualize both the living and dead/membrane-compromised bacteria with fluorophores SYTO 9 (final concentration of 2 μg/mL; S34854; Thermo Fisher Scientific) and PI (final concentration of 25 μg/mL), respectively. Depending on the treatment group, microbubbles (final concentration of 10^7^ microbubbles/mL) were added at the same time. The OptiCell was orientated with the biofilm on the upper membrane and mixed to achieve homogenous distribution. For the microbubbles to float up to the biofilm and any planktonic bacteria to fall down, as well as dye incubation, 5 min were allowed to elapse for every biofilm before experiments. Each OptiCell was moved along the X and Y directions, while taking care that each new area was not acoustically overlapping with at least 1 cm margins. Time-lapse confocal laser scanning microscopy was performed for 10 min using three channels: (1) SYTO 9 excited at 488 nm, detected at 525/50 nm (center wavelength/bandwidth); (2) PI excited at 561 nm, detected at 595/50 nm; and (3) DiD excited at 640 nm, detected at 700/75 nm. Each field-of-view consisted of 512 × 512 pixels (128 × 128 μm) and was imaged at 1.29 frames per second using bi-directional, resonant scanning. Optical image recordings began 15 s prior to insonification to establish the initial cell state and continued for 585 s to detect bacterial responses to the different exposure conditions. Any focus drift that occurred was manually corrected during the imaging procedure.

### 2.7. Fluorescence Image Analysis

Post hoc image analysis was performed in MATLAB (The MathWorks, Natick, MA, USA) on the time-lapse images acquired with the confocal microscope. The fluorescence intensity was monitored in the control groups to ensure that time-lapse imaging did not lead to phototoxicity or bleaching. Particle counting was performed using custom-designed analysis software to determine the total number of cells in a field-of-view, as a quantitative measure of dispersion and sonoporation using the channels for SYTO 9 and PI imaging (Figure 1C,D). Images were first converted to binary by thresholding (threshold for SYTO 9 at 600 and PI at 800; image intensity ranging from 0–4095). Next, all connected components were identified through the bwconncomp function in MATLAB. All connected components with an area between 0.375 and 1.25 µm^2^ were counted as individual particles, and those larger than 1.25 µm^2^ were subsequently watershed for proper image segmentation of the constituent particles. The output of the image segmentation was visualized with a color-coded map of the counted particles (Figure 1E). 

The initial number of cells was defined as those counted in the frame with the highest SYTO 9 intensity before ultrasound (the first 15 s of recording). The number of particles after treatment was defined as the mean number of particles counted in three frames, recorded after ultrasound by selecting the frame with maximum SYTO intensity within the intervals between 1–2, 5–6, and 9–10 min. We chose to select these four single frames, with maximum SYTO 9 intensity per recording, to achieve quantification in the same Z-plane, thereby ensuring that the results were unaffected by focus drift. For sonoporation, two measures were used to assess changes in PI as a response to treatment. First, the change in PI, relative to only the initial PI amount, was calculated:

(1) $$PI\;Change\;\left(\%\right)=\left(PI\frac{after}{\;initial}-1\right)\times 100$$

Second, the relative increase in the number of PI cells was considered, in the context of all cells in a field-of view, by using the following formula:

(2) $$Sonoporation\;\left(\%\right)=\left({\left(\frac{\left[PI\right]}{\left[SYTO\;9\right]}\right)}_{after}-{\left(\frac{\left[PI\right]}{\left[SYTO\;9\right]}\right)}_{initial}\right)\times 100$$

For dispersion, the removal of cells from the field-of-view was calculated with:

(3) $$Dispersion\;\left(\%\right)=\left(\frac{{\left(PI+SYTO\;9\right)}_{initial}-{\left(PI+SYTO\;9\right)}_{after}}{{\left(PI+SYTO\;9\right)}_{initial}}\right)\times 100$$

For microbubble displacement, we quantified the change in microbubble distribution across the field-of-view, since the high amount of displacement results in microbubble clustering. We identified all areas of adjacent microbubbles by using a similar particle-tracking algorithm, as described previously in this section for particle counting. For this, the DiD channel was thresholded at 800, and all connected components identified with an area larger than 3.1 µm^2^ (corresponding to a microbubble radius of 1 µm). The change in the number of identified areas is a measure for microbubble clustering—before ultrasound, a high number of areas are identified as corresponding to individual microbbubles; after the microbubble displacement caused by ultrasound, only a few areas will be identified as corresponding to large clusters of microbubbles. Microbubble clustering was calculated with the following formula:

(4) $$Microbubble\;Clustering\;\left(\%\right)=\left(\frac{{\left(DiD\right)}_{initial}-{\left(DiD\right)}_{after}}{{\left(DiD\right)}_{initial}}\right)\times 100$$

### 2.8. Statistics

Quantifiable data are graphically displayed in box plots, which show the median, interquartile range, and whiskers from minimum to maximum or a dot plot. Outliers from the data are indicated with a black dot, based on whether the data point was above or below 1.5× the interquartile range. The statistical analysis of the acquired data was performed using MATLAB and GraphPad Prism 8 (GraphPad Software Inc., San Diego, CA, USA), with the assumption that the quantitative data was non-normally distributed. To determine significant differences among the various acoustical settings, with and without microbubbles, as well as whether the addition of oxacillin to the same treatment group had significance, the Mann–Whitney U nonparametric test was used on the output of Equations (1)–(3) for each individual experiment. In total, 176 individual experiments were conducted, from which, the n number, per a treatment group, is located on the top of the box plots for each group. A result was considered significant when the *p* value was found to be <0.05. 

## 3. Results

### 3.1. Isolate Characterization and Biofilm Confirmation

**Figure 2:**
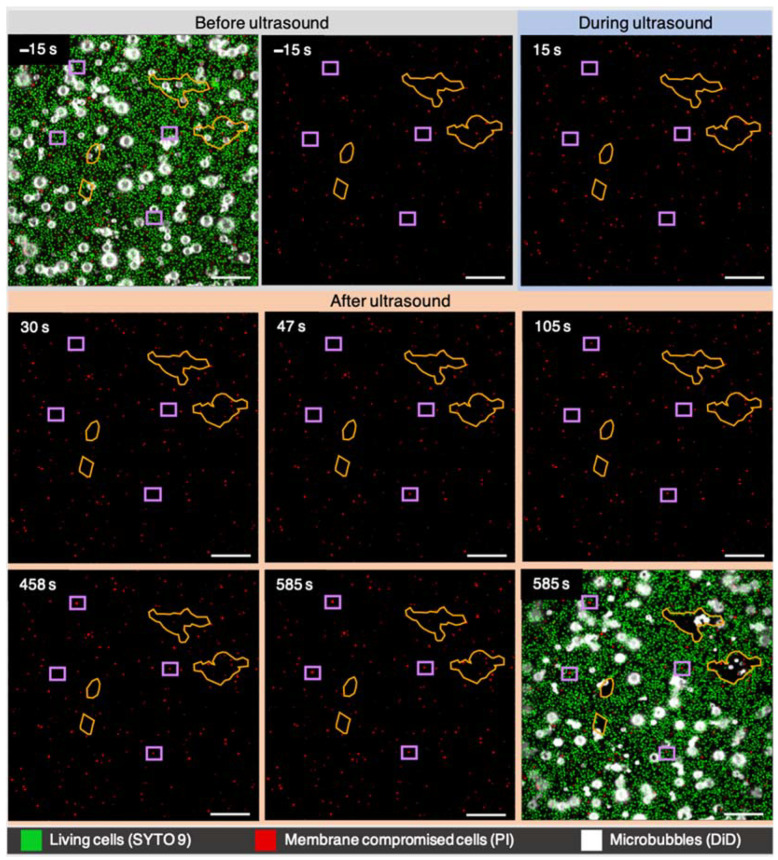
Sonobactericide of *S. aureus* biofilm at ultrasound settings of 2 MHz, 200 kPa, 100 cycles, PRF 1 Hz, 30 s. Selected confocal microscopy frames during a 10 min time-lapse recording, where the first and last frames are a composite of all three channels, and all others are the PI channel only. Time 0 s is the start of insonification. Purple squares highlight example cells with PI uptake at different timepoints, and orange outlines mark regions of dispersion. Scale bars represent 20 μm.

**Figure 3:**
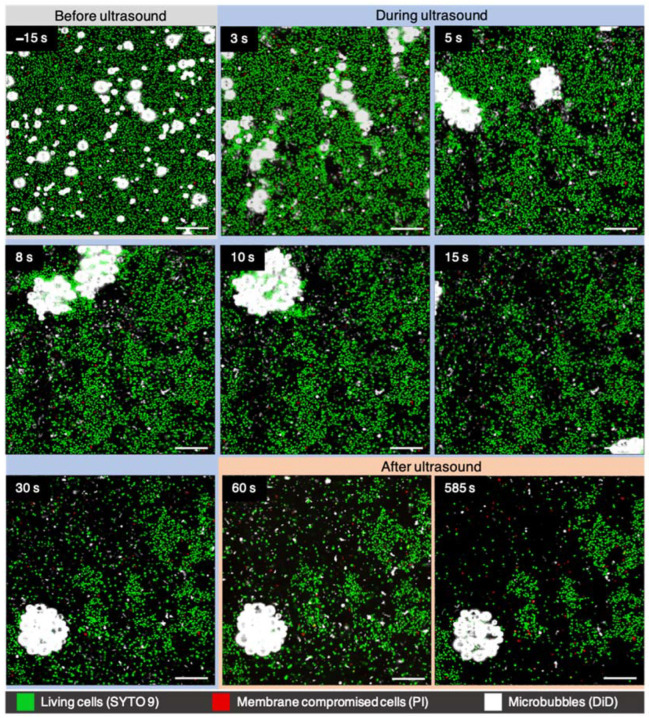
Sonobactericide of *S. aureus* biofilm at ultrasound settings of 2 MHz, 400 kPa, 100 cycles, PRF 1 Hz, 30 s. Selected confocal microscopy frames during a 10 min time-lapse recording of all three channels together. Scale bars represent 20 μm.

The clinical isolate was identified as a *S. aureus* strain with a genetic background belonging to sequence-type (ST) 398 and *spa*-type t571. The isolate used in this study was found to be methicillin-sensitive, with a minimal inhibitory concentration of ≤0.25 μg/mL for oxacillin. Further susceptibility was found for all other antibiotics tested, except for erythromycin and clindamycin. Sequencing established biofilm-producing capability and the presence of various virulence factor genes, which can be found in Supplementary Table S1. Fluorescence staining (Figure 1C) confirmed a biofilm mass covering the OptiCell membranes. Confocal microscopy using live/dead staining confirmed a bacterial population within the biofilms, composed of predominately viable bacterial cells (Figure 2 and Figure 3). The total amount of bacterial cells, on average, in a single field-of-view was 7105 ± 1580 (average ± SD). Minimal photobleaching and phototoxicity was observed over the full time-lapse imaging procedure for all three fluorophores and subsequently confirmed with fluorescence intensity monitoring of the imaging control experiments (no ultrasound insonification and with or without microbubbles). 

### 3.2. Sonobactericide

Figure 2 shows an example of a biofilm treated with sonobactericide at 200 kPa and 100 cycles. Bacteria with noticeable PI uptake were observed across the entire field-of-view and occurred throughout the 555 s, following the 30 s insonification. This is highlighted by the purple squares in Figure 2, showing examples where a cell became PI-positive at 47 s (i.e., 17 s after insonification) in the bottom square, 105 s (i.e., 75 s after insonification) in the top square, 458 s (i.e., 428 s after insonification) in the right square, and 585 s (i.e., 555 s after insonification) in the left square. At this setting, bacterial dispersal was minimal (main areas outlined in orange). Most ultrasound-induced microbubble displacement led to dispersion. The attractive movements of microbubbles toward each other leading to cluster formation, and the clusters moving, as a whole, led to the most noticeable dispersion. All outlined dispersion areas involved microbubbles <4 um, and the two largest areas had the most microbubbles clustering together (5, uppermost outline; 6, second uppermost). Lipid shedding was observed directly with cluster formation and microbubble movement (see Supplementary Video S1). 

PI uptake also occurred during and throughout the recording, following insonification at the higher pressure setting of 400 kPa, with the same number of cycles, namely 100 (Figure 3). By the first imaging frame, ~500 ms after ultrasound began, microbubble clustering could already be seen, and lipid shedding and fragmentation could be seen by the second frame (~800 ms) (Supplementary Video S2). Both SYTO 9 and PI-positive cells were seen to be displaced and/or dispersed by microbubble movements. Most microbubbles in the field-of-view clustered, which occurred faster and led to far more dispersion than what was observed at lower pressures, as seen in Figure 2. The large cluster in the bottom three frames of Figure 3 consisted of more than 20 microbubbles, and, altogether, a width of 33.8 μm at the largest point. Microbubbles touching or with the size 4.5 ± 0.5 μm (average ± SD; min 3.8 μm–max 5.4 μm) prior to insonification were seen to be the drivers of clustering. The majority of solitary ≤2 μm microbubbles did not displace to form clusters, but remained non-displaced, fragmented, or were swept up by cluster movement. By 5 min of imaging, most liberated bacterial movement, due to dispersal events, had settled and lipid debris levels stabilized. Some lipid debris remained throughout the entirety of the recording, after having already been formed by microbubble lipid shedding and fragmentation by the first US pulse. 

**Figure 4:**
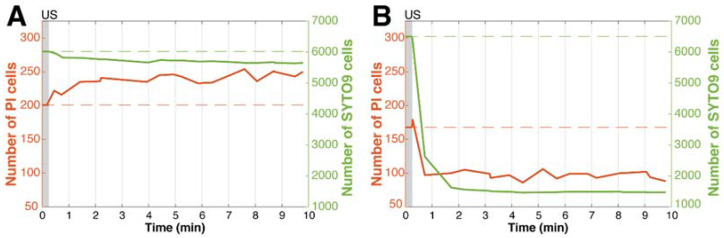
Different rates of dispersion and sonoporation of bacteria within biofilms. Example time-lapse line graphs during and after ultrasound with microbubbles treatment indicating (**A**) minimal dispersion and increasing propidium iodide uptake (ultrasound: 2 MHz, 200 kPa, 100 cycles, PRF 1 Hz, 30 s), corresponding to Figure 2, and (**B**) high dispersion with minimal propidium iodide uptake (ultrasound: 2 MHz, 400 kPa, 100 cycles, PRF 1 Hz, 30 s), corresponding to Figure 3. The grey bar at time point 0 indicates the 15 s before ultrasound insonification. (PI = propidium iodide; US = ultrasound).

The biofilm responses to the two different ultrasound settings, seen in the confocal microscopy examples (Figure 2 and Figure 3), can be further understood when quantifying the bacterial population based on fluorescence of cell-state separately, as shown in Figure 4. For the lower pressure setting (Figure 4A), the number of PI-positive cells increased by 26.4%, while the living cells stained with SYTO 9 decreased by 6.4%. By doubling the pressure, but keeping the cycles the same, a dramatically different outcome transpired, which can be seen in Figure 4B. PI uptake initially shows a spike in PI-positive cells, followed by a sharp decrease of 52.0%; then, it continues to stay around this percentage for the remainder of imaging. The number of SYTO 9 cells experienced a more dramatic decrease of 77.5%, which took 3 min to stabilize, due to dispersed cell movements still ongoing after microbubble-induced dispersion, as seen in Figure 3. Both graphs show higher rates of dispersal for SYTO 9 cells than PI-positive cells, even when considering that the true PI-positive cell counts dispersed is slightly offset by PI uptake that occurred at the same time.

**Figure 5:**
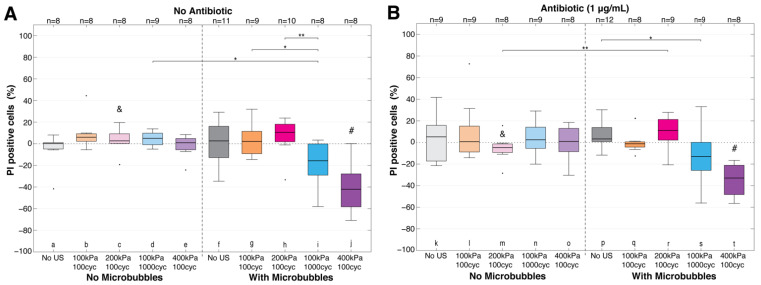
Sonoporation considering change in propidium iodide-positive cells only (Equation (1); PI change (%)). Key statistically significant differences are indicated above the box plots with * *p* < 0.05 or ** *p* < 0.01. Statistical significance from all other groups within the same graph are marked by a (#). If adding an antibiotic, (**B**) was different from the same treatment without (**A**), this is denoted with (&) for *p* < 0.05. Black circles denote outliers. cyc = cycles; PI = propidium iodide; US = ultrasound. See Supplementary Table S2 for all statistical comparisons.

Figure 5 focuses on sonoporation by examining the PI uptake independent of dispersion and from all experimental data beyond the two examples (Figure 2 and Figure 3). Without an antibiotic (Figure 5A), all treatment settings without microbubbles (a–e) were not significantly different from each other or the no ultrasound with microbubbles group (f). With microbubbles, the lowest pressure setting with 100 cycles (Figure 5A (g)) was not different from all treatment groups without microbubbles or without ultrasound (Figure 5A (a–f)). The number of PI-positive cells decreased significantly for the two higher settings (Figure 5A (i,j)) when compared to the majority of the other experimental arms, including the same ultrasound settings without microbubbles, i.e., 100 kPa, 1000 cycles, and 400 kPa, 100 cycles. With the addition of an antibiotic (Figure 5B), the variability in treatment responses changed compared to Figure 5A. The treatment outcomes with microbubbles and an antibiotic were found to not be significantly different from microbubbles without an antibiotic. The changes in variability altered significance patterns; 100 kPa, 1000 cycles (Figure 5B (s)) was no longer significantly different from experimental groups without microbubbles (Figure 5B (k–o)), yet still different from all other microbubbles treatment groups (Figure 5B (q,r,t)); as well as 200 kPa, 100 cycles (Figure 5B (r)) gaining significance over the same setting without microbubbles (Figure 5B (m)) and to the same number of cycles but half the pressure with microbubbles (Figure 5B (q)) (i.e., 100 kPa, 100 cycles). The two higher ultrasound settings with an antibiotic (Figure 5B (s,t)) also showed decreases in overall PI-positive cells as seen without an antibiotic (Figure 5A (i,j)).

**Figure 6:**
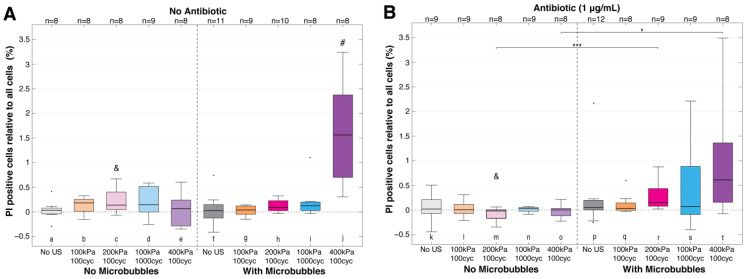
Sonoporation relative to all cells in field-of-view (Equation (2). Sonoporation (%)). Key statistically significant differences are indicated with * *p* < 0.05 or *** *p* < 0.001. Black circles denote outliers. Statistical significance from all other groups within the same graph are marked by a (#). If adding an antibiotic (**B**) was different from the same treatment without (**A**), that is denoted with (&) for *p* < 0.05. cyc = cycles; PI = propidium iodide; US = ultrasound. See Supplementary Table S3 for all statistical comparisons.

**Figure 7:**
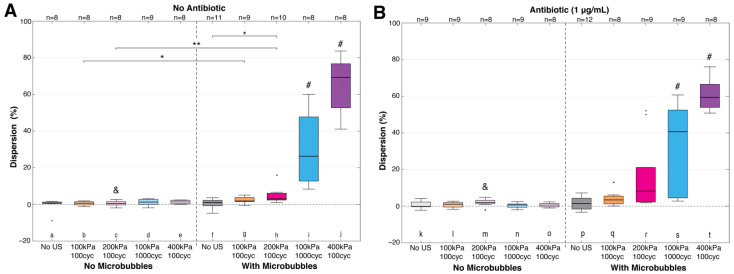
Dispersion of bacteria within biofilms (Equation (3); dispersion (%)). Key statistically significant differences are indicated with * *p* < 0.05. or ** *p* < 0.01. Black circles denote outliers. Statistical significance from all other groups within the same graph are marked by a (#). If adding an antibiotic, (**B**) was different from the same treatment without (**A**), which is denoted with (&) for *p* < 0.05. cyc = cycles; US = ultrasound. See Supplementary Table S4 for all statistical comparisons.

In order to understand the complete story, the total PI uptake was placed in the context of the ~7000 cells present in a field-of-view and accounting for dispersion (Figure 6). The higher acoustic pressure of 400 kPa, 100 cycles with microbubbles (Figure 6A (j)), showed a significantly higher, more variable response, with an increase of PI uptake up to 3.2%, despite having the highest percentage of biofilm removal (Figure 7). For the other settings, the overall change in PI-positive cells was close to negligible, with <1% for all treatment groups without an antibiotic (Figure 6A). When the antibiotic was included in the experiments (Figure 6B), the outcome was also <1% for the tested groups without microbubbles (k–o), with microbubbles without ultrasound (p), and for microbubbles with ultrasound at 100 and 200 kPa with 100 cycles (q, r). Increases in acoustic pressure or cycles with microbubbles and antibiotic resulted in more PI-positive cells (Figure 6B (r–t)). Half the pressure, but 10× the cycles (100 kPa, 1000 cycles, Figure 6B (s)), led to significantly more PI uptake (up to a maximum of 2.2%), with a wider value range than without an antibiotic, despite the significantly higher rate of dispersion seen with an antibiotic at this setting (Figure 7). The highest pressure setting of 400 kPa, 100 cycles with microbubbles and an antibiotic resulted in a lower median PI uptake than with an antibiotic at the same setting, yet had the largest amount of PI-positive cells (up to 3.5%). 

Figure 7 takes both cell populations (SYTO 9 and PI) into account to determine the total amount of dispersion. For all settings without microbubbles and no antibiotic (Figure 7A (a–e)), minimal dispersion (≤3.1%) occurred. This was similar for microbubbles and no ultrasound (≤3.7%) (Figure 7A (f)). With ultrasound, microbubbles, and each successive parameter increase (Figure 7A (g–j)), the number of dispersed cells dramatically increased for the two higher ultrasound settings, with up to a maximum of 83.7% biofilm removal at 400 kPa. At 100 cycles, the difference between 100 and 200 kPa (Figure 7A (g,h)) was not significant, but the addition of microbubbles was significant for both, compared to its ultrasound-only control without microbubbles (Figure 7A (b,c)). A higher significance was also found for the higher ultrasound settings (Figure 7A (i,j)), against their no microbubble controls, which also extended to all other experimental groups (Figure 7A (a–h)), with a much higher level of variability in dispersal response. Antibiotic presence generally led to more dispersed cells for all groups with microbubbles, except for 400 kPa, 100 cycles, which had 9.9% less dispersal (average). Variability in responses decreased for 400 kPa, 100 cycles, when an antibiotic was added (Figure 7B (t)), but led to a wider range of values for the other microbubble treatment groups (Figure 7B (q–s)).

**Figure 8:**
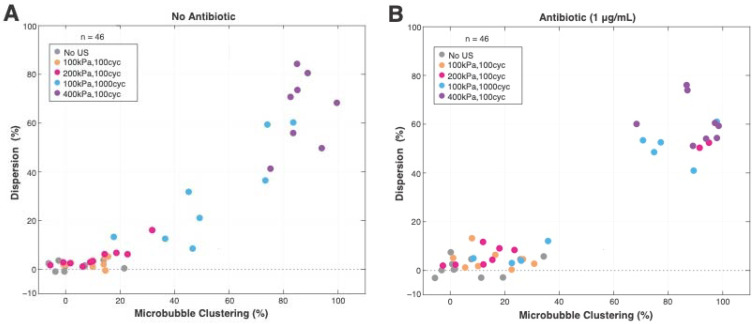
Microbubble clustering used as a measure of microbubble displacement corresponding to bacterial dispersion (Equation (4); microbubble clustering (%)) following insonification without an antibiotic (**A**) and with an antibiotic (**B**) present. cyc = cycles; US = ultrasound.

The number of microbubbles displacing into clusters corresponded to the amount of bacterial dispersion (Figure 8). The more microbubbles clustered and, thus, displaced, the more dispersion that occurred. Regarding all treatment groups with and without an antibiotic (Figure 8A,B), the highest pressure setting (400 kPa) led to the highest amount of microbubble clustering (68.1–99.8%), and 85.1% clustering at 400 kPa resulted in the most dispersion (83.7%). The lowest pressure, but highest cycles (100 kPa, 100 cycles), had the most variable clustering response (17.8–83.8%), though generally held the direct relationship with dispersion (Figure 8A). The addition of an antibiotic resulted in a less variable response, where <40% clustering had <20% dispersion, and >68% clustering had 40–80% dispersion (Figure 8B). Regarding sonoporation, there was no direct correlation found with our measure for microbubble displacement. Instead, sonoporation only correlated with the higher ultrasound pressure and cycle settings, as seen in Figure 5 and Figure 6.

## 4. Discussion

To the best of our knowledge, this is the first study that investigated the direct effects of sonobactericide, in terms of dispersion and sonoporation in real-time and at high-resolution. A *S. aureus* clinical isolate was used to produce biofilms that were subsequently exposed to ultrasound with or without microbubbles. The addition of an antibiotic was also used to evaluate synergism, in response to sonobactericide. During continuous high-resolution fluorescence microscopy over 10 min, dispersion was quantified by bacterial cells leaving the field-of-view and sonoporation by cellular PI uptake. 

Based on published literature, both the 398 sequence and t571 *spa*-type of the clinical isolate used in this study have been implicated in several types of invasive infections, including blood stream, intravascular device-associated, and post-cardiac surgery infections, and they were found in other countries outside of the Netherlands and Europe [38,39,40,41,42,43,44,45]. The t571 *spa*-type designation with human-associated ST398 has been observed repeatedly and is sometimes disproportionally more common than other *spa*-types [38,41,42]. Additionally, the methicillin-sensitive ST398 variant (including with t571) is suggested to be more virulent than both methicillin-resistant ST398 and -sensitive non-ST398 strains, as well as associated with a higher 30-day mortality [38,39]. 

Sonoporation, defined as membrane permeabilization, as a result of ultrasound and oscillating microbubbles, was observed by PI fluorescence upon and after treatment. The bacteria that showed immediate PI uptake was most likely more susceptible to and/or experienced more microbubble-induced effects. This could be location-dependent, in relation to the microbubble, or enhanced, due to the physiological state of the cell, or other endo- or exogenous processes. For cells that took longer to become PI-positive, the microbubble-induced damage and stress experienced was most likely not enough to cause direct cell membrane impairment, and the activated repair mechanisms were sufficient at preventing immediate lysis. To determine the cell fate of the bacteria, PI uptake cannot be used in the same manner as for mammalian cells. In mammalian cells, if the PI uptake stabilizes after 120 s, the cell is considered irreversibly sonoporated, ultimately leading to cell death [14]. Optical observations in this study show PI uptake occurring instantaneously within one frame and already at maximum intensity. Though sonoporation can readily be seen in single mammalian cells as a transient and increasing PI influx lasting over minutes [10,14], this may not occur in bacteria, or a fluorophore binding threshold or certain resolution needs to be met for visualization. The latter seems plausible, given the optical set-up in this study, which had lateral and axial resolutions of up to 200 and 600 nm [46], respectively. The pore size necessary for a single bacterium to not immediately lyse would need to be in the magnitude of 1–10 nm, since a 100 nm pore is already a tenth of the cell size. In mammalian cells, membrane pores as small as 1 nm have been observed with electron microscopy, following microbubble-induced sonoporation [47]. Since a pore would only need to be at least the diameter of a PI molecule, which is 1.3 nm [48], the limitation to conclusively know whether transient PI uptake can occur in *S. aureus* then lies with the optical detection limit. Therefore, the PI uptake curves cannot be extrapolated for bacteria, and we cannot further conclude from our study whether sonoporation was irreversible, leading to cell death, or reversible. Cell death, whether that be apoptosis or necrosis, and irreversible and reversible sonoporation, as a consequence of sonobactericide, remains to be determined and should be investigated in future studies using supportive, alternative assays.

Control experiments were performed to negate PI uptake that was potentially due to natural, spontaneous cellular responses that were not driven by sonobactericide. Ultrasound alone did not induce significant cell death and is supported by studies that found only microbubble cavitation to induce significant stress responses [49] or microstructure damage [50] in bacteria. It is possible that the effects of sonoporation may take more than 585 s to induce cell death, and a longer imaging protocol might have detected more PI-positive cells, similar to other sonobactericide papers, which waited 24 h after ultrasound before optical imaging [50,51]. The identification of delayed cell death may be missed when assessing viability at one specific timepoint or time-window, thus underestimating the true impact of sonobactericide. Though this is outside the scope of this study, it remains an important consideration for future investigations, since the complete cell response pathways for microbubble-induced bacterial damage, stress, or death are unknown, along with whether a bacterium can survive once triggered.

Sonoporation at higher pressures and cycles is challenging to track on a single-cell level, due to the high rates of dispersion observed in this study. For the two higher ultrasound settings (Figure 5A (i,j) and Figure 5B (s,t)), decreases in the overall PI-positive cells were found for all four treatments, which is attributed to the loss of cells, due to dispersion at these setting (Figure 6A (i,j) and Figure 6B (s,t)). However, this only reveals part of the story, since the cellular responses of the bacteria that were dispersed or fell off are unknown. Existing evidence suggests that bacteria released from biofilms using chemical dispersal agents may be more virulent than the typical planktonic cells for a few hours before becoming highly susceptible again [52,53,54]. It is possible that the mechanical microbubble-induced disruption of the biofilm does not lead to this more virulent profile, and/or many are damaged in the process that leads to immediate or delayed cell lysis. This is supported by Wille et al., who observed that mechanically dispersed bacteria may not alter bacteria, such as that of chemical dispersal agents [55]. Thus, cells dispersed by sonobactericide should be fully characterized, in order to determine and address the potential safety issues and challenges that these cells, whether more virulent or not, could pose; thus, this will be the subject of our future studies.

The polydisperse microbubbles employed in this study had a mean diameter of 4 μm, which is slightly larger than the current commercially available, lipid-shelled contrast agents (1.1–3.6 μm [56]). However, other sonobactericide studies used similarly sized microbubbles [50,51,57], and the mean diameter was in range of the clinically approved human albumin-coated microbubbles (Optison, mean diameter (range) 3–4.5 μm). It is of potential importance that this larger size is closer to resonance frequency, at 2 MHz [58], which could translate to a more efficacious sonobactericide treatment, since a larger microbubble population would achieve maximum excursion amplitude. The microbubble concentration used in this study was chosen to maximize sonoporation potential, due to the sheer number of cells (~7000), and was supported by Dong et al. [50], who observed that a higher microbubble concentration led to enhanced bacterial PI uptake in biofilms. On the one hand, a lower concentration may enhance dispersal by minimizing the shielding effect that can happen with higher microbubble concentrations and larger bubble sizes at higher pressures [59,60]. Shielding could have contributed to the heterogenous responses seen in some settings. On the other hand, enhanced microbubble displacement may result in less cell death, as reported in endothelial cells, where non-displacing microbubbles were more lethal [14]; although, it should be noted, again, that mammalian cells and *S. aureus* are very different cell types. To further understand the relationship between microbubble displacement and bacterial cell death and dispersion, a similar study, such as that of van Rooij et al. [14], could be conducted using non-targeted and targeted microbubbles for bacterial biofilms, such as with the innovative, recently published targeted microbubbles using vancomycin [17] or Affimer protein [25]. Bacterial dispersal was a direct consequence of microbubble displacement and subsequent clustering, quantitatively supported in Figure 8, which was possibly due to secondary Bjerknes forces. Our data is in agreement with the current knowledge that clustering happens quickly (ms) [61]. The range of microbubble sizes (3.8–5.4 μm) leading to clustering were both within, and slightly outside, of resonance; however, that was determined at 50 kPa [58]. Increasing pressure is known to (linearly) dampen resonance frequency [62]. 

The ultrasound-induced microbubble dynamics leading to dispersal that were observed in this study explain the previous high-resolution sonobactericide paper findings of biofilm disruption and craters, since these were not observed in real-time and only after treatment (>5 min–24 h) [23,50,51,63]. Craters and cell detachment have been described in confluent cervical cancer cell monolayers, but such phenomena required high-pressure (4 MPa, peak-negative pressure), shock-wave bubble cluster generation [64]. Around the detachment area, Ohl et al. [64] observed a rim of dead cells (the time between ultrasound exposure and observational imaging was not provided). In our study, PI-positive bacterial cells were found throughout the entire field-of-view, and a higher number was not observed, particularly around the rim of the dispersal areas. Concerning microbubble behavior beyond displacement and clustering, acoustic cavitation was not monitored, so it cannot be concluded in this study whether stable or inertial cavitation was responsible for the effects seen at the different ultrasound settings. Future studies employing passive cavitation monitoring or ultra-high-speed imaging will help to elucidate which microbubble responses, as well as the ensuing biological, physical, and chemical effects, are most advantageous for treating biofilm infections with sonobactericide.

Antibiotic synergism was mildly observed in this study, as seen with more variable responses, more dispersion at some settings, and a slightly higher percentage of PI-positive cells, when considering the whole field-of-view. This effect was neither significant nor as sharply observed as in other sonobactericide studies looking into therapeutic synergism [23,50,63]. This could be due to the different ultrasound parameters, bacterial strain/species, or experimental design and set-up. This study utilized a shorter insonification time that did not destroy all the microbubbles by the end of treatment, as well as an experimental set-up, which minimized standing wave formation. Another consideration is that the recording length may be too short to capture the long-term effect, as supported by Figure 4A, which that shows increasing PI uptake over the 10 mins. Future studies could include real-time imaging, followed by hourly sampling points, to address this. Another possibility is that the 3.5 h waiting step after adding oxacillin may also lead to an underestimation. This step was performed to better understand the effects that were microbubble-induced and not directly from the antibiotic alone. It is possible that performing the experiments 0.5 h after antibiotic addition could have a more synergistic effect on sonobactericide. This further highlights the importance of experimental design and antibiotic pharmacodynamics, which should be explored, and the in vivo situation considered in future studies. These considerations would prevent under- or overestimating treatment outcomes, as well as determine, for clinical translation, if sonobactericide is best administered together with a fresh antibiotic dose for maximal effect. 

## 5. Conclusions

Both bacterial cell dispersion and sonoporation were observed in this investigative study on sonobactericide. Dispersion was more prominent than sonoporation, as a result of the oscillating non-targeted microbubbles for *S. aureus* biofilms, as well as with the acoustical settings of 2 MHz, 100–400 kPa, 100–1000 cycles, every s for 30 s. Increasing the acoustic pressure had more of an effect on dispersion and sonoporation than increasing the number of cycles. Antibiotic presence led to more variable treatment responses and did not significantly enhance sonobactericide, suggesting the synergism reported by others is not an immediate effect. To conclude, these findings provide informative details, in real-time and at high-resolution, on how sonobactericide can directly aid in biofilm infection treatment. 

## Data Availability

All data presented in this study are available on request from the corresponding author.

## Supplementary Materials

The following supporting information can be downloaded at https://www.mdpi.com/article/10.3390/pharmaceutics14061164/s1. Table S1: Genes present in the infective endocarditis *S. aureus* isolate. Table S2: Statistical comparisons of sonoporation corresponding to Figure 5. Table S3: Statistical comparisons of relative sonoporation corresponding to Figure 6. Table S4: Statistical comparisons of dispersion corresponding to Figure 7. Video S1: Time-lapse recording of a *S. aureus* biofilm exposed to sonobactericide at ultrasound settings of 2 MHz, 200 kPa, 100 cycles, PRF 1 Hz, 30 s, corresponding to Figure 2. Video S2: Time-lapse recording of a *S. aureus* biofilm exposed to sonobactericide at ultrasound settings of 2 MHz, 400 kPa, 100 cycles, PRF 1 Hz, 30 s, corresponding to Figure 3.
