# Supplementary material for: Dispersing and Sonoporating Biofilm-Associated Bacteria with Sonobactericide
Source: Pharmaceutics. 2022 May 30;14(6):1164. doi: 10.3390/pharmaceutics14061164 (PMC9227517; doi:10.3390/pharmaceutics14061164)
Supplement: Supplementary file 1 [file pharmaceutics-14-01164-s001.zip › pharmaceutics-1685697-supplementary/Supplementary/Supplemental_Tables.pdf]

Supplemental Information

Manuscript: Dispersing and Sonoporating Biofilm-associated Bacteria with Sonobactericide

Kirby R. Lattwein [1], Inés Beekers [1], Joop J.P. Kouijzer [1], Mariël Leon-Grooters [1], Simone A.G. Langeveld [1], Tom van Rooij [1], Antonius F.W. van der Steen [1,2], Nico de Jong [2,1], Willem J.B. van Wamel [3], and Klazina Kooiman [1]

1 Department of Biomedical Engineering, Thoraxcenter, Erasmus University Medical Center, Rotterdam, the Netherlands

2 Laboratory of Medical Imaging, Department of Imaging Physics, Delft University of Technology, Delft, the Netherlands

3 Department of Medical Microbiology and Infectious Diseases, Erasmus University Medical Center, Rotterdam, the Netherlands

Supplemental Table S1. Genes present in the infective endocarditis *S. aureus* isolate.

**Gene (clonal complex-associated)**		**Gene (biofilm-associated)**	
arc	carbamate kinase	agrB	transmembrane endopeptidase; quorum sensing regulator
aroE	shikimate dehydrogenase	fnbA	fibronectin binding proteins; high biofilm production association
glpF	glycerol kinase	fnbB	
gmk	guanylate kinase	clfA	clumping factors; cell surface-associated protein responsible for fibrinogen binding and human platelet aggregation
pta	phosphate acetyltransferase	clfB	
tpi	triosephosphate isomerase	fib	extracellular fibrinogen binding protein
yqil	acetyl coenzyme A acetyltransferase	ebps	elastin binding protein
**Gene (biofilm-associated)**		eno	laminin binding protein
icaA	intercellular adhesions; biofilm producing ability, PIA synthesizer	cna	collagen binding protein
icaD		RNAIII	detergent-like peptides; PSMs
icaB		sarA	transcription regulator of biofilm formation, development, and several virulence factors
icaC			

PSMs = phenol-soluble modulins, PIA = polysaccharide intercellular adhesion

Supplemental Table S2A. Statistical comparisons of sonoporation corresponding to Figure 5A.

No Antibiotic		No Microbubbles					With Microbubbles				
		No US	100kPa, 100cyc	200kPa, 100cyc	100kPa, 1000cyc	400kPa, 100cyc	No US	100kPa, 100cyc	200kPa, 100cyc	100kPa, 1000cyc	400kPa, 100cyc
No Microbubbles	No US		0.0728	0.1282	0.2810	0.7104	0.8601	>0.9999	0.0079	0.0401	0.0022
	100kPa, 100cyc	0.0728		>0.9999	0.6943	0.2086	0.9298	0.4698	0.2105	0.0059	0.0006
	200kPa, 100cyc	0.1282	>0.9999		0.6126	0.3178	0.7914	0.6806	0.2991	0.0022	0.0003
	100kPa, 1000cyc	0.2810	0.6943	0.6126		0.5358	0.9039	0.6058	0.0927	0.0104	0.0006
	400kPa, 100cyc	0.7104	0.2086	0.3178	0.5358		0.9298	0.8371	0.0311	0.0541	0.0022
With Microbubbles	No US	0.8601	0.9298	0.7914	0.9039	0.9298		0.8820	0.3312	0.0908	0.0018
	100kPa, 100cyc	>0.9999	0.4698	0.6806	0.6058	0.8371	0.8820		0.2224	0.0464	0.0010
	200kPa, 100cyc	0.0079	0.2105	0.2991	0.0927	0.0311	0.3312	0.2224		0.0016	0.0002
	100kPa, 1000cyc	0.0401	0.0059	0.0022	0.0104	0.0541	0.0908	0.0464	0.0016		0.0499
	400kPa, 100cyc	0.0022	0.0006	0.0003	0.0006	0.0022	0.0018	0.0010	0.0002	0.0499	

Supplemental Table S2B. Statistical comparisons of sonoporation corresponding to Figure 5B.

Antibiotic (1 µg/mL)		No Microbubbles					With Microbubbles				
		No US	100kPa, 100cyc	200kPa, 100cyc	100kPa, 1000cyc	400kPa, 100cyc	No US	100kPa, 100cyc	200kPa, 100cyc	100kPa, 1000cyc	400kPa, 100cyc
No Microbubbles	No US		>0.9999	0.4559	0.7304	0.9626	0.5538	>0.9999	0.4363	0.4363	0.0006
	100kPa, 100cyc	>0.9999		0.3450	0.5414	0.8785	0.2083	0.7789	0.1672	0.1672	0.0002
	200kPa, 100cyc	0.4559	0.3450		0.1810	0.6620	0.0135	0.0565	0.0076	0.2721	0.0027
	100kPa, 1000cyc	0.7304	0.5414	0.1810		0.4807	0.7682	0.8371	0.4363	0.1135	0.0002
	400kPa, 100cyc	0.9626	0.8785	0.6620	0.4807		0.5714	0.6943	0.2879	0.1672	0.0006
With Microbubbles	No US	0.5538	0.2083	0.0135	0.7682	0.5714		0.1249	0.5198	0.0278	<0.0001
	100kPa, 100cyc	>0.9999	0.7789	0.0565	0.8371	0.6943	0.1249		0.0756	0.0712	0.0003
	200kPa, 100cyc	0.4363	0.1672	0.0076	0.4363	0.2879	0.5198	0.0756		0.1359	0.0002
	100kPa, 1000cyc	0.4363	0.1672	0.2721	0.1135	0.1672	0.0278	0.0712	0.1359		0.0152
	400kPa, 100cyc	0.0006	0.0002	0.0027	0.0002	0.0006	<0.0001	0.0003	0.0002	0.0152	

Supplemental Table S3A. Statistical comparisons of relative sonoporation corresponding to Figure 6A.

No Antibiotic		No Microbubbles					With Microbubbles				
		No US	100kPa, 100cyc	200kPa, 100cyc	100kPa, 1000cyc	400kPa , 100cyc	No US	100kPa, 100cyc	200kPa, 100cyc	100kPa, 1000cyc	400kPa, 100cyc
No Microbubbles	No US		0.2284	0.0813	0.2238	0.9497	0.4923	0.8639	0.1471	0.1375	0.0007
	100kPa, 100cyc	0.2284		0.9591	0.8884	0.4418	0.1220	0.1388	0.6965	0.3357	0.0003
	200kPa, 100cyc	0.0813	0.9591		0.8884	0.2786	0.0545	0.0927	0.5726	0.6126	0.0019
	100kPa, 1000cyc	0.2238	0.8884	0.8884		0.4807	0.0947	0.1615	0.7197	0.6065	0.0025
	400kPa, 100cyc	0.9497	0.4418	0.2786	0.4807		0.6965	0.9626	0.6965	0.6943	0.0011
With Microbubbles	No US	0.4923	0.1220	0.0545	0.0947	0.6965		0.5490	0.0524	0.1331	<0.0001
	100kPa, 100cyc	0.8639	0.1388	0.0927	0.1615	0.9626	0.5490		0.2110	0.1416	<0.0001
	200kPa, 100cyc	0.1471	0.6965	0.5726	0.7197	0.6965	0.0524	0.2110		0.8868	<0.0001
	100kPa, 1000cyc	0.1375	0.3357	0.6126	0.6065	0.6943	0.1331	0.1416	0.8868		0.0003
	400kPa, 100cyc	0.0007	0.0003	0.0019	0.0025	0.0011	<0.0001	<0.0001	<0.0001	<0.0001	

Supplemental Table S3B. Statistical comparisons of relative sonoporation corresponding to Figure 6B.

Antibiotic (1 µg/mL)		No Microbubbles					With Microbubbles				
		No US	100kPa, 100cyc	200kPa, 100cyc	100kPa, 1000cyc	400kPa, 100cyc	No US	100kPa, 100cyc	200kPa, 100cyc	100kPa, 1000cyc	400kPa, 100cyc
No Microbubbles	No US		0.9314	0.4234	0.7962	0.6730	0.6038	0.7577	0.0770	0.6048	0.0274
	100kPa, 100cyc	0.9314		0.1996	0.7962	0.6058	0.4967	0.8371	0.0400	0.5457	0.0206
	200kPa, 100cyc	0.4234	0.1996		0.1672	0.3823	0.0085	0.0721	0.0003	0.2359	0.0148
	100kPa, 1000cyc	0.7962	0.7962	0.1672		0.4234	0.1333	0.7577	0.0056	0.5457	0.0360
	400kPa, 100cyc	0.6730	0.6058	0.3823	0.4234		0.1220	0.3357	0.0079	0.3704	0.0281
With Microbubbles	No US	0.6038	0.4967	0.0085	0.1333	0.1220		0.4173	0.0947	0.9682	0.0545
	100kPa, 100cyc	0.7577	0.8371	0.0721	0.7577	0.3357	0.4173		0.0229	0.8371	0.0939
	200kPa, 100cyc	0.0770	0.0400	0.0003	0.0056	0.0079	0.0947	0.0229		0.6048	0.2766
	100kPa, 1000cyc	0.6048	0.5457	0.2359	0.5457	0.3704	0.9682	0.8371	0.6048		0.2359
	400kPa, 100cyc	0.0274	0.0206	0.0148	0.0360	0.0281	0.0545	0.0939	0.2766	0.2359	

Supplemental Table S4A. Statistical comparisons of dispersion corresponding to Figure 7A.

No Antibiotic		No Microbubbles					With Microbubbles				
		No US	100kPa, 100cyc	200kPa, 100cyc	100kPa, 1000cyc	400kPa, 100cyc	No US	100kPa, 100cyc	200kPa, 100cyc	100kPa, 1000cyc	400kPa, 100cyc
No Microbubbles	No US		0.5358	0.3969	0.7577	0.6943	0.9298	0.0311	0.0033	0.0003	0.0003
	100kPa, 100cyc	0.5358		>0.9999	0.4234	0.1949	0.5999	0.0360	0.0025	0.0002	0.0002
	200kPa, 100cyc	0.3969	>0.9999		0.4807	0.2786	0.4920	0.0592	0.0016	0.0002	0.0002
	100kPa, 1000cyc	0.7577	0.4234	0.4807		>0.9999	0.9408	0.1615	0.0315	<0.0001	<0.0001
	400kPa, 100cyc	0.6943	0.1949	0.2786	>0.9999		0.8404	0.2766	0.0079	0.0002	0.0002
With Microbubbles	No US	0.9298	0.5999	0.4920	0.9408	0.8404		0.1519	0.0159	<0.0001	<0.0001
	100kPa, 100cyc	0.0311	0.0360	0.0592	0.1615	0.2766	0.1519		0.2581	<0.0001	<0.0001
	200kPa, 100cyc	0.0033	0.0025	0.0016	0.0315	0.0079	0.0159	0.2581		<0.0001	<0.0001
	100kPa, 1000cyc	0.0003	0.0002	0.0002	<0.0001	0.0002	<0.0001	<0.0001	<0.0001		0.0047
	400kPa, 100cyc	0.0003	0.0002	0.0002	<0.0001	0.0002	<0.0001	<0.0001	<0.0001	0.0047	

Supplemental Table S4B. Statistical comparisons of dispersion corresponding to Figure 7B.

Antibiotic (1 µg/mL)		No Microbubbles					With Microbubbles				
		No US	100kPa, 100cyc	200kPa, 100cyc	100kPa, 1000cyc	400kPa, 100cyc	No US	100kPa, 100cyc	200kPa, 100cyc	100kPa, 1000cyc	400kPa, 100cyc
No Microbubbles	No US		>0.9999	0.0907	0.7304	>0.9999	0.4639	0.0549	0.0052	0.0005	<0.0001
	100kPa, 100cyc	>0.9999		0.0549	0.4363	0.6730	0.6511	0.0907	0.0079	<0.0001	<0.0001
	200kPa, 100cyc	0.0907	0.0549		0.0052	0.0205	0.6504	>0.9999	0.2086	0.0033	0.0003
	100kPa, 1000cyc	0.7304	0.4363	0.0052		0.8884	0.5538	0.0311	0.0012	<0.0001	<0.0001
	400kPa, 100cyc	>0.9999	0.6730	0.0205	0.8884		0.5208	0.0401	0.0037	<0.0001	0.0002
With Microbubbles	No US	0.4639	0.6511	0.6504	0.5538	0.5208		0.3845	0.0683	0.0018	<0.0001
	100kPa, 100cyc	0.0549	0.0907	>0.9999	0.0311	0.0401	0.3845		0.2086	0.0115	0.0003
	200kPa, 100cyc	0.0052	0.0079	0.2086	0.0012	0.0037	0.0683	0.2086		0.0311	0.0003
	100kPa, 1000cyc	0.0005	<0.0001	0.0033	<0.0001	<0.0001	0.0018	0.0115	0.0311		0.0055
	400kPa, 100cyc	<0.0001	<0.0001	0.0003	<0.0001	0.0002	<0.0001	0.0003	0.0003	0.0055	
